# Explaining the Correlates of Eating Outside-of-Home Behavior in a Nationally Representative US Sample Using the Multi-Theory Model of Health Behavior Change: A Cross-Sectional Study

**DOI:** 10.3390/ijerph21010115

**Published:** 2024-01-20

**Authors:** Manoj Sharma, Christopher Johansen, Ravi Batra, Chia-Liang Dai, Sidath Kapukotuwa, Bertille Assoumou, Kavita Batra

**Affiliations:** 1 Department of Social and Behavioral Health, School of Public Health, University of Nevada, Las Vegas, NV 89119, USA; manoj.sharma@unlv.edu (M.S.); christopher.johansen@unlv.edu (C.J.); kapukotu@unlv.nevada.edu (S.K.); 2 Department of Internal Medicine, Kirk Kerkorian School of Medicine at UNLV, University of Nevada, Las Vegas, NV 89102, USA; 3 Department of Environmental and Occupational Health, School of Public Health, University of Nevada, Las Vegas, NV 89119, USA; ravi.batra123@gmail.com; 4 Department of Teaching and Learning, College of Education, University of Nevada, Las Vegas, NV 89154, USA; chia-liang.dai@unlv.edu; 5 Department of Surgery, Kirk Kerkorian School of Medicine at UNLV, University of Nevada, Las Vegas, NV 89102, USA; 6 Office of Research, Kirk Kerkorian School of Medicine at UNLV, University of Nevada, Las Vegas, NV 89102, USA; 7 Department of Medical Education, Kirk Kerkorian School of Medicine at UNLV, University of Nevada, Las Vegas, NV 89102, USA

**Keywords:** multi-theory model, screening, eating outside home behavior, eating away from home meals

## Abstract

Eating outside-of-home (EOH) is one of the main changes in lifestyle that occurred worldwide in the past few decades. Given that EOH behavior is influenced by individual and contextual factors, the utilization of a theory seems to be suitable in analyzing this health behavior. The fourth-generation theory multi-theory model (MTM) is designed exclusively for health behavior change at the individual and community levels. Therefore, the purpose of this analytical cross-sectional study was to investigate EOH behavior by using the MTM among a nationally representative sample in the United States (US). Data for this study were collected from April–May 2023 via a 61-item psychometric valid, web-based, structured survey disseminated via Qualtrics. Chi-square/Fisher’s exact tests were used to compare categorical data, whereas the independent-samples *t*-test was used to compare the mean scores of MTM constructs across groups. Pearson correlation analysis was performed for the intercorrelation matrix between the MTM constructs and hierarchical regression models were built to predict the variance in the initiation and sustenance by certain predictor variables beyond demographic characteristics. The *p* values in the multiple comparisons were calculated by using adjusted residuals. Among a total of 532 survey respondents, 397 (74.6%) indicated being engaged in EOH at least twice a week, whereas 135 (25.4%) reported not being engaged in EOH. People who were engaged in EOH were younger (mean age = 42.25 ± 17.78 years vs. 55.89 ± 19.43 years) African American, (15.9% vs. 6.7%, *p* = 0.01), single or never married, (34.0% vs. 23.0%, *p* = 0.02), had a graduate degree (9.6% vs. 3.7%, *p* = 0.03), and were employed (72.0% vs. 34.8%, *p* < 0.001) as opposed to those who reported not being engaged in eating outside the home. Among the MTM constructs of initiation, “behavioral confidence” and “changes in the physical environment” were the significant predictors of initiating a reduction in EOH behavior and explained 48% of the variance in initiation. Among the MTM constructs of sustenance, “emotional transformation” and “changes in the social environment” were the significant predictors of sustaining a reduction in EOH behavior and explained 50% of the variance in sustenance. This study highlights a need to design MTM-based educational interventions that promote in-home eating instead of frequent EOH for health, family bonding, economic, and other reasons.

## 1. Introduction

Eating outside-of-home (EOH) commonly refers to consuming food at a restaurant or fast food [1]. Other terms used for EOH include: away-from-home meals, out-of-home food consumption, takeaway food, ready-to-eat, ala carte, cafeteria, buffet, bar, takeaway, and cafes [1]. EOH is a common lifestyle behavior in modern society. EOH is one of the main changes in lifestyle that occurred worldwide in the past few decades [1]. This lifestyle change is greatly influenced by individual, cultural, socioeconomic, social, biological, environmental, and psychological factors [1]. In the United States (US), recent evidence suggests that over half of American adults report three or more away-from-home meals per week and over 35% report two or more fast food meals per week [2]. EOH is fairly consistent across income and age groups; for instance, households with income above 300% of the federal poverty guidelines report consuming food away from home 5.5 times per week, whereas households with income at or below 300% of the federal poverty guidelines report consuming food away from home 4.8 times per week [3].

Previous research has consistently shown that EOH is associated with an increased intake of calories, saturated fat, and sodium, lower consumption of macronutrients (i.e., vitamin C, iron, calcium, and fiber), and lower consumption of fruits and vegetables [1,4,5], combined with a large portion size, which may result in weight gain, obesity, and insulin resistance [5,6]. Research suggests an association with increased daily total energy and fat intake for restaurants and staff/school canteens, as well as an association with higher intake of sodium for restaurants [7]. A higher intake of beverages, such as soft drinks, sugar-sweetened beverages, fruit juices, beer, and other alcohol, is associated with EOH [1]. Furthermore, in a cross-sectional analysis of an ancillary dataset from the Survey of Health of Wisconsin, which was collected in six Wisconsin counties as part of the Centers for Disease Control and Prevention Community Transformation Grant, researchers reported a positive association with body mass index (BMI) with every one meal/week at a fast-food and sit-down restaurant, and a positive association with EOH at these restaurants with the unavailability of healthy foods at shopping and eating venues, and lack of cooking skills [8]. Thus, this demonstrates a need to better understand the correlates of EOH.

Recent evidence suggests that sociodemographic factors such as sex, race/ethnicity, education, income, and BMI status have been associated with EOH [9]. Full-time employment of mothers and single parents is also inversely correlated with EOH [5]. Preparing food at home is reported to be less convenient compared with EOH. Convenience is positively correlated with EOH [5], and available time to prepare food at home is inversely correlated with EOH [10]. 

Given that EOH behavior is influenced by individual and contextual factors, the utilization of a theory seems to be useful in analyzing this health behavior [11]. Research investigating dietary behaviors incorporating a theoretical framework to understand the correlates of EOH is needed [12]. The fourth-generation multi-theory model (MTM) is designed exclusively for health behavior change at the individual and community levels. The MTM attempts to investigate both short-term and long-term changes through two components: initiation and sustenance of a specific health behavior [13]. MTM components and each construct have been tested to explain a variety of health behaviors and are applicable to populations across their lifespan [14,15,16,17,18,19].

The component of initiation in the MTM entails three constructs: “participatory dialogue”, “behavioral confidence”, and “changes in the physical environment”, seeking to explain the start of the behavior change [13]. Participatory dialogue emphasizes the individual’s exploration of the perceived advantages and perceived disadvantages of behavior change. Behavioral confidence can derive from internal (e.g., craving) and external (e.g., peer) sources. Changes in the physical environment involve the accessibility and attainability of resources to create behavioral change. Unlike other behavioral change theories/models that focus on learning behavior, the unique feature of the MTM is its inquiry into the maintenance of behavior change for the long term. The component of sustenance in the MTM also consists of three constructs, which are emotional transformation, practice for change, and changes in the social environment that are necessary for maintaining the behavior [13]. Emotional transformation means converting feelings (e.g., fear) to goals toward changing the behavior. Practice for change refers to reflective action, such as reflecting on the behavior change regularly, adapting coping strategies, and overcoming barriers. Changes in the social environment can be seeking support from friends, family, and professionals.

The current study seeks to explore the potential of the MTM in explaining EOH behaviors. The utilization of the MTM as a framework is also crucial for developing effective evidence-based interventions to enhance healthy eating habits. The MTM has been successfully applied to assess various eating-related behaviors (e.g., fruit and vegetable consumption, portion size, and water consumption) across diverse populations [18,19,20]. Based on the findings of these studies, we hypothesized that the constructs of the initiation and sustenance models within the MTM support the explanation of EOH behaviors. Specifically, the study aims to investigate EOH behavior using the MTM among a nationally representative sample in the US.

## 2. Materials and Methods

### 2.1. Study Design and Participants

Through this analytical cross-sectional study, a nationally representative (by gender, age, region, household income, and race/ethnicity) sample of US adults aged 18 years or above was recruited. Only English-speaking participants were included. The study samples were obtained from currently available pools of research participants who had consented to be contacted for future studies. To avoid heavy dependence on a single segment of the population, Qualtrics pooled samples from different sources across the nation. The enforced quota constraints closely matched the U.S. Census data, as shown below in Table 1.

### 2.2. Data Collection

Data for this study were collected from April–May 2023 via a psychometric valid, web-based, structured survey disseminated via Qualtrics (Provo, UT, USA) survey collection tool. Data were collected with the help of the research marketing team within the Qualtrics company, which helps researchers to collect high-quality data from even hard-to-reach population groups. The Qualtrics team disseminates surveys among their panel providers through a variety of methods, including listserv and/or in-app notifications. The Qualtrics team also helps in the initial testing of the survey by sending the survey to some potential respondents, and this process is termed as the “soft launch”. This soft launch is a good proxy for the pilot or initial testing of the survey in which researchers can identify any issues with the survey items, as well as with data prior to the full launch or broader dissemination. This study was conducted in accordance with the Declaration of Helsinki principles to allow voluntary and well-informed participation from the potential respondents. All data were de-identified and efforts were made to avoid selection bias by posing screening questions at the beginning of the survey. Eligible participants who completed the survey were offered incentives in a variety of forms (e.g., gift cards, SkyMiles, cash rewards, and/or redeemable points), as outlined by the contract between the Qualtrics marketing research team and its panel providers.

### 2.3. Questionnaire

**Figure 1:**
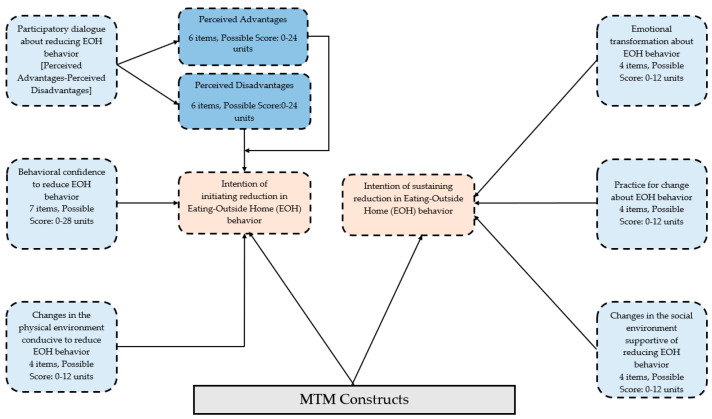
MTM framework for changing eating-outside-home (EOH) behavior.

The theoretical framework used for this 61-item survey tool was the MTM [15,21]. The MTM has two components of “initiation” and “sustenance”, which were used as dependent variables in this study and were measured by a single item on a 5-point Likert scale ranging from ‘not at all likely’ to ‘completely likely.’ Initiation has three constructs, namely “perceived advantages”, “perceived disadvantages”, “behavioral confidence”, and “changes in the physical environment”. When the score of “perceived disadvantages” is subtracted from the score of “perceived advantages”, it results in a derived construct of “participatory dialogue”. Similarly, sustenance also has three constructs, namely “emotional transformation”, “practice for change”, and “changes in the social environment”. All details about this MTM-based tool are shown in Figure 1 below. 

### 2.4. Survey Validation

**Figure 2:**
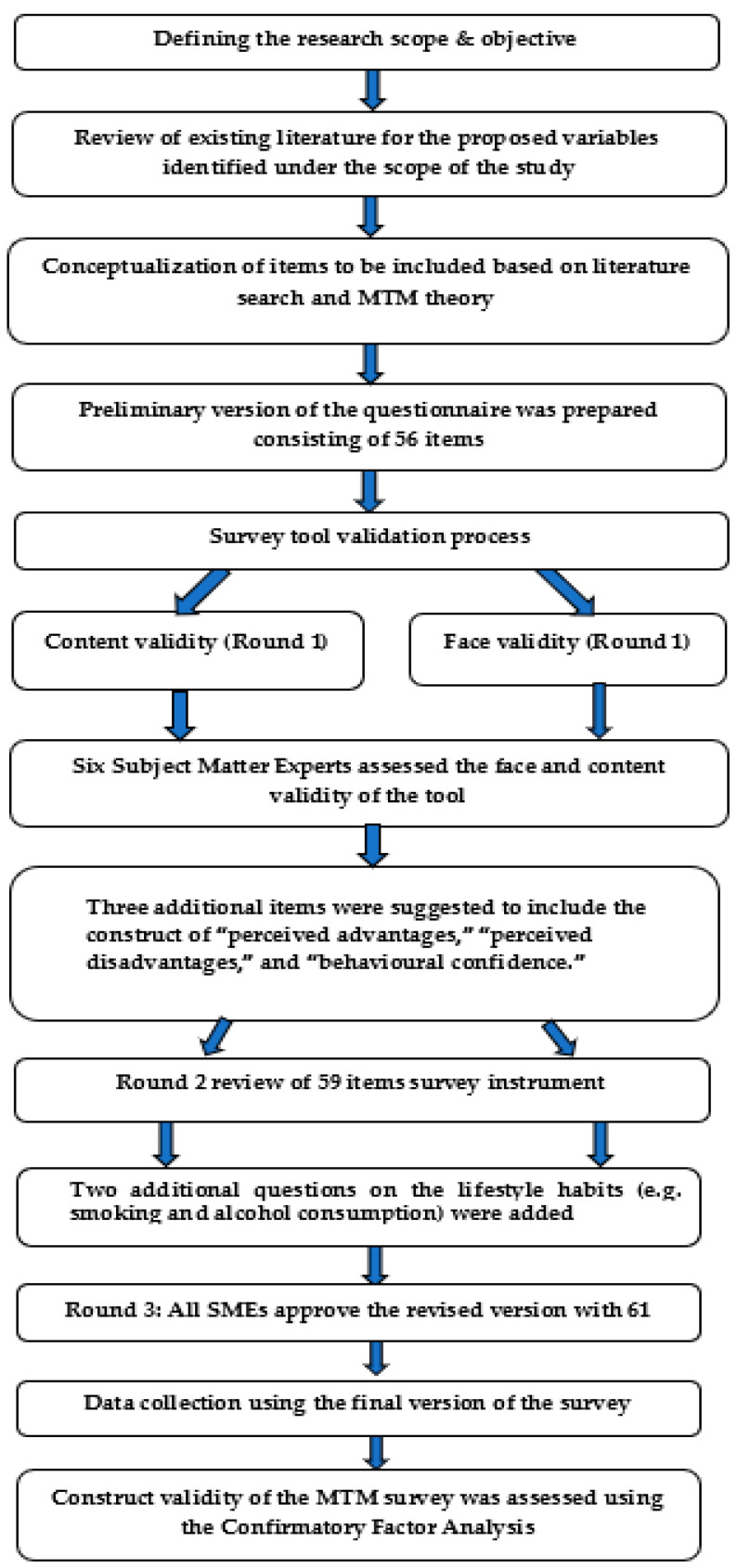
Flowchart detailing all steps of establishing the validity of the survey tool.

The survey underwent face and content validation by six subject matter experts (of which four were university faculty with multiple expert areas) in the fields of multi-theory model (*n* = 5), nutrition (*n* = 2), target population familiarity (*n* = 6), and instrument validation (*n* = 4). Since the experts had multiple expertise, the numbers listed in the parentheses above may not sum to a total of six. The initial version included a total of 56 items. Experts provided comments to establish the face validity, content validity, and qualitative construct validity of the instrument and improve the readability/clarity/formatting of the survey, and five additional items were included, one each for advantages, disadvantages, and behavioral confidence, and two for demographics (which were moved toward the end), in the survey. The rest of the changes entailed some wordsmithing to improve the readability for the target population. There was consensus among experts after three rounds to arrive at the final 61-item instrument (Figure 2). 

### 2.5. Structural Equation Modeling (Construct Validity)

We used the Mplus 8.4 software package and robust weighted least squares (WLSMV) as the estimator to test the measurement model. Model fit was assessed using the comparative fit index (CFI) with values above 0.90 [22], and the root-mean-square error of approximation (RMSEA) less than 0.08 as evidence of acceptable fit [23].

### 2.6. Statistical Analysis

First, the univariate analysis was performed to describe the data and also to identify any patterns in the data. Categorical variables were represented as counts and proportions, whereas continuous variables were reported as means and standard deviations, unless otherwise stated. The box plot was inspected to assess outliers in the data. The assumption of normality was assessed by Shapiro–Wilk’s test (*p* > 0.05). Chi-square/Fisher’s exact tests were used to compare categorical data, whereas the independent-samples *t*-test was used to compare the mean scores of the MTM constructs across groups. Pearson correlation analysis was performed for the intercorrelation matrix between the MTM constructs and hierarchical regression models were built to predict the variance in the initiation and sustenance by certain predictor variables beyond demographic characteristics. For the tables more than 2 by 2 in the chi square, the exact *p* values were calculated by using adjusted residuals. For the independent-samples *t*-test, Levene’s test for equality of variance was conducted to check the assumption of the homogeneity of variance. We also ran hierarchical multiple regression to predict the initiation (continuous variable) by a series of models, including demographic characteristics, and MTM constructs. This was to determine the R-square change and improvement in prediction after adding variables during the model-building process. A similar hierarchical model was built with sustenance as a dependent variable too. Prior to running the regression models, linearity was assessed by partial regression plots and a plot of standardized residuals against the predicted values. The independence of residuals was assessed by the Durbin–Watson statistics. The multicollinearity was assessed by the tolerance and variance inflation factor (VIF). We also performed multinominal logistic regression to model the log odds of the initiation and sustenance levels. There was a total of 5 levels, including “not at all likely (0), “somewhat likely” (1), “moderately likely” (2), “very likely” (3), and “completely likely” (4). We further recoded these levels to a total of three levels by combining “not at all likely” with “somewhat likely” and by merging “very likely” with “completely likely”. For the reliability diagnostics, we calculated Cronbach’s alpha, as well as McDonald’s Omega. The 95% confidence intervals of proportions were calculated by the normal approximation to the binomial calculation. IBM SPSS (V.28) was used to analyze the data, and the level of significance was set at 5%.

### 2.7. A-Priori Power Analysis

For a priori power analysis, we used the following formula:

n = p ×⋅q × (zα/2E)^2^, (1)

where p and q (1-p) are taken as 0.35 and 0.65, the margin of error (E) = 0.05, and Z value = 1.96 (which corresponds to the 95% confidence level). 

The proportion (p) of 35% stated above was based on evidence by An (2016) that suggested that over 35% U.S. individuals reported two or more fast food meals per week [9]. The minimum sample required was 350 and, after accounting for 20% non-response, the final sample was 420, which was larger than the sample used in this study to allow structural equation modeling [24]. 

## 3. Results

### 3.1. Structural Equation Modeling (Measurement Model)

**Figure 3:**
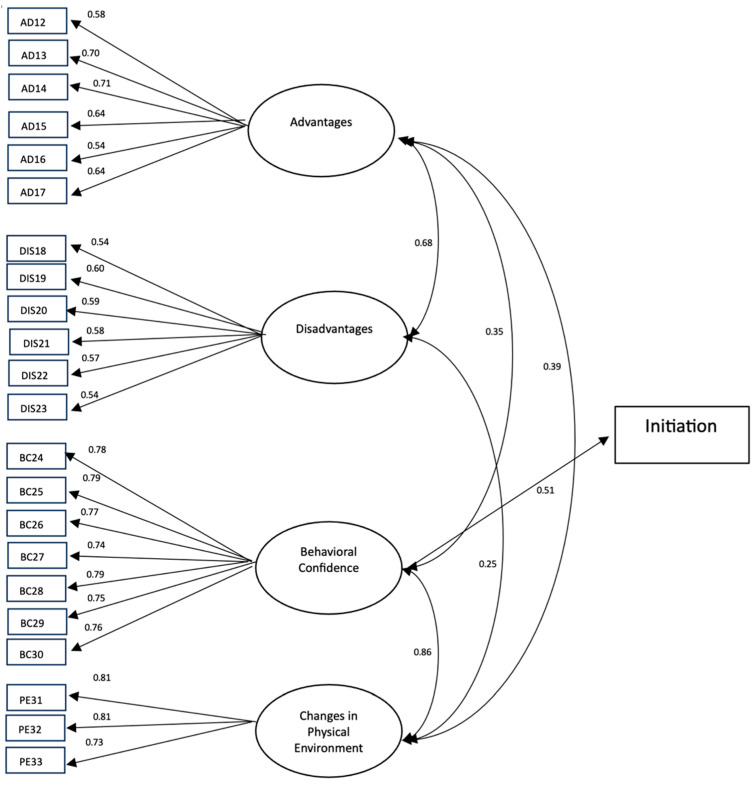
A measurement model for initiating a reduction in EOH behavior. Note: Only factor loadings with statistical significance are shown in this figure.

**Figure 4:**
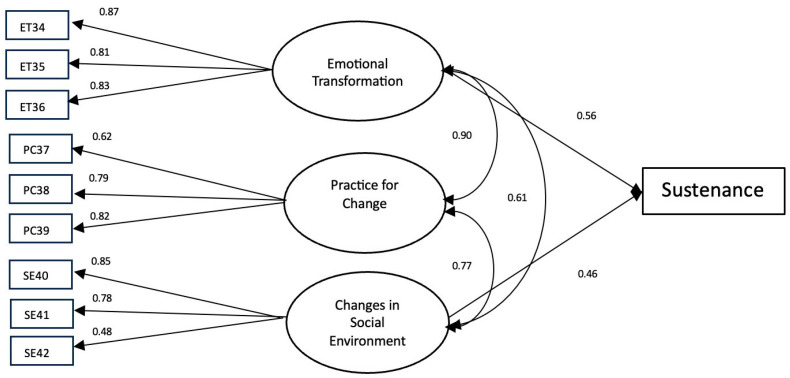
A measurement model of sustaining a reduction in EOH behavior. Note: Only factor loadings with statistical significance are shown in this figure.

The results of the initiation model yielded an estimated RMSEA of 0.06 while missing the conventional thresholds for the CFI index, but only marginally (0.89). The standardized factor loadings ranged from 0.54 to 0.81 (Figure 3), which indicated that the initiation scale provided valid measurement of its constructs (i.e., perceived advantages (ADs), perceived disadvantages (DISs), behavioral confidence (BC), and changes in physical environment (PE). For the sustenance model, the results indicated that the model fitted the data well, with values of CFI 0.97 and RMSEA 0.05. The standardized factor loadings in the sustenance scale ranged from 0.48 to 0.87 (Figure 4). These effects suggested that the sustenance scale also provided a valid measurement of its constructs (i.e., emotional transformation (ET), practice for change (PC), and changes in social environment (SE)).

### 3.2. Comparing Baseline Characteristics and MTM Scores among Groups

Among a total of 532 survey respondents or participants, 397 (Group 1: 74.6%) indicated being engaged in eating outside the home at least twice a week, whereas 135 (Group 2: 25.4%) indicated that they were not eating outside home at least twice a week (Table 2). People who were engaged in EOH were younger (mean age = 42.25 ± 17.78 years vs. 55.89 ± 19.43 years), Black or African American (15.9% vs. 6.7%, *p* = 0.01), single or never married (34.0% vs. 23.0%, *p* = 0.02), had a graduate degree (9.6% vs. 3.7%, *p* = 0.03), and were employed (72.0% vs. 34.8%, *p* < 0.001, Table 2) as opposed to those who reported not being engaged in the eating outside the home. On the contrary, a significantly larger proportion of those who were not eating outside the home at least twice a week was from the Northeast region (22.2% vs. 13.6%, *p* = 0.02, Table 2) as opposed to those engaged in eating outside the home behavior. Upon comparing eating patterns and other behavioral characteristics, it was found that, among those who reported eating outside the home at least twice a week, a significantly larger proportion reported being current smokers (34.8% vs. 20.7%, *p* = 0.002) and eating in fast-food restaurants (40.6% vs. 25.9%, *p* < 0.001) as opposed to the group 2 (Table 3). People eating outside the home were spending a significantly larger amount of money as opposed to group 2. As shown in Table 4, the mean scores of the intention of initiating and sustaining a reduction in the EOH behavior were higher among those not engaged in this behavior, with statistically significant mean differences (*p* < 0.001). Likewise, the mean scores of “behavioral confidence”, “changes in the physical environment”, “emotional transformation”, and “practice for change” were higher among those not engaged in this behavior, with statistically significant mean differences (*p* < 0.001, Table 4). On the contrary, the mean scores of “perceived disadvantages” of reducing EOH were higher among those who were already practicing or engaged in the behavior (Group 1). 

### 3.3. Bivariate Correlation and Reliability

As indicated in Table 5, all values of Cronbach’s alpha and McDonald’s omega were over 0.70, with a maximum value of 0.910, which corresponds to excellent reliability. The construct of behavioral confidence was moderately to strongly and positively correlated with all other MTM constructs, with the exception of the participatory dialogue. Please see all intercorrelation matrices in Table 5.

### 3.4. Findings of Regression Analyses

For the dependent variable of the initiation, the results of hierarchical multiple regression indicated that people who were engaged in EOH behavior had a 0.661 points lower score of intention (initiation) of reducing this behavior. Among the MTM constructs of initiation, “behavioral confidence” and “changes in the physical environment” were significant predictors of initiating a reduction in EOH behavior. All predictors in the regression equation explained 48% (as denoted by the adjusted R^2^ in Table 6) of the variance in the initiation. For the dependent variable of sustenance, the results of hierarchical multiple regression indicated that people who are engaged in EOH behavior have a 0.600 points lower score of intention (sustenance) of reducing this behavior. Among the MTM constructs of sustenance, “emotional transformation” and “changes in the social environment” were the significant predictors of sustaining a reduction in EOH behavior. All predictors in the regression equation explained 50% (as denoted by the adjusted R^2^ in Table 7) of the variance in sustenance. Additionally, people living in the West region had a 0.310 points lower score of intention (sustenance) of reducing this behavior as opposed to those living in the Midwest region. Notably, with each unit increase in behavioral confidence, participants with an intention of initiating a reduction in EOH had higher odds by 1.1 units (*p* = 0.004) of falling in the “moderately likely” category and 1.3 units higher odds of falling in the “very likely” category (Table 8). Also, with each unit increase in “changes in the physical environment”, participants with an intention of initiating a reduction in the EOH had higher odds by 1.33 units (*p* < 0.001) of falling in the “very likely” category (Table 8). Participants who were already engaged in EOH behavior had 74% lower odds of falling in the “very likely” category. Consistent with the results of the hierarchical regression’s findings, people living in the western region had 66.2% (*p* = 0.04) lower odds of falling in the “very likely” category (Table 8). Likewise, with each unit increase in emotional transformation, participants with an intention of sustaining a reduction in EOH had higher odds by 1.24 units (*p* = 0.003) of falling in the “moderately likely” category and 1.75 units higher odds of falling in the “very likely” category (Table 9). Also, with each unit increase in “changes in the social environment”, participants with an intention of sustaining a reduction in the EOH had higher odds by 1.28 units (*p* < 0.001) of falling in the “moderately likely” category and 1.46 units higher odds of falling in the “very likely category” (Table 9). Participants who were already engaged in the EOH behavior had 69.4% lower odds of falling in the “moderately likely” category and 82.3% lower odds of falling in the “very likely” category for sustenance. Consistent with the results of the hierarchical regression’s findings, people living in the Western region had 74.5% (*p* = 0.04) lower odds of falling in the “very likely” category (Table 9). Younger age was also associated with lower odds of being in the “moderately likely” and “very likely” categories of sustaining a reduction in EOH behavior (Table 9).

## 4. Discussion

This study aimed at examining the theory-based correlates of eating outside-of-home (EOH) behavior in a US sample. It was found that approximately three-quarters of our sample ate outside of the home for at least two days of the week. The percentage of fast-food consumption among those engaged in EOH for more than two weeks was significantly higher than that of those eating fewer than two times per week outside of the home. The proportion of those eating outside of the home is very high and those eating fast foods has also increased substantially in comparison with the NHANES data from 2007–2010, which reported about 35% of American adults consuming two or more fast-food meals per week [2]. Furthermore, people who were eating outside of the home were relatively younger. For self-evident reasons, the amount of money spent on EOH was significantly higher among those who ate outside of the home on more than two occasions per week. This increasing trend of EOH behavior is somewhat alarming, given the variety of negative consequences for a person’s health, as well as home economics. This highlights the need for health promotion programs to be developed to reduce EOH behavior and encourage the cooking of meals at home, especially among youth.

Upon descriptive examination of the MTM constructs between the two groups of people EOH at least twice a week and those not EOH twice a week, the directionality of all MTM constructs was in the theory-predicted direction, except for perceived advantages; however, it was not statistically significant. This could be attributed to the temptingness of the taste, convenience, and type of foods as putative causes of allurement to EOH [5,10,24], which may have played a role in lowering the advantages score.

The main conclusion of the inferential work in the initiation model using hierarchical regression modeling was that two constructs of MTM, namely behavioral confidence (predicted value of intent of reducing EOH increased by 0.08 units for each unit of behavioral confidence) and changes in the physical environment (0.09 unit increase) along with EOH (predicted value of intent 0.66 units lower than that of those who did not), were statistically significant, and accounted for 48% of the variance in reducing EOH behavior. This proportion of predicted variance is considered quite high in behavioral and social sciences [21]. Upon further examination of the initiation model using multinomial regression, it was found that there was a greater odds of those likely to start changing their EOH behavior when compared with those not likely to change of 1.37 (95% CI: 1.14, 1.56) for changes in the physical environment and 1.31 (95% CI: 1.22, 1.42) for behavioral confidence. These findings lend support to the predictive potential of these two constructs to initiate change in decreasing the frequency of EOH behavior. While no studies have been conducted with this model and EOH behavior, other studies utilized the MTM that supports the role of these two constructs in dietary behaviors [18,19,20]. Further studies with EOH have shown that a lack of cooking skills leads to indulging in this behavior, which is in line with the finding of our study that behavioral confidence was a significant correlate of initiating change in EOH behavior [8]. Likewise, proximity to fast-food restaurants has also been found to be an important factor for EOH [2], which is in line with our study’s finding of changes in the physical environment as a salient construct in influencing the initiation of EOH change. Health promotion interventions must reify these constructs to promote in-home eating and reduce EOH behavior (see Implications for Practice). 

The main conclusion of the inferential work in the sustenance model using hierarchical regression modeling was that two constructs of MTM, namely emotional transformation (predicted value of intent increases by 0.16 units for each unit of emotional transformation) and changes in the social environment (0.11 units increase) along with EOH (0.60 units lower than those who did not) and the region being Western US (0.31 units lower compared with the Midwest) were statistically significant and accounted for 50% of the variance in reducing EOH behavior. This proportion of predicted variance is considered quite high in behavioral and social sciences [21]. Upon further examination of the sustenance model using multinomial regression, it was found that there was a greater odds of being likely to maintain changing their EOH behavior when compared with those not likely to change as 1.47 (95% CI: 1.29, 1.67) for changes in the social environment and 1.76 (95% CI: 1.48, 2.09) for emotional transformation. While no studies have been conducted with this model and EOH behavior, other studies have been with the MTM that supports these two constructs in dietary behaviors [18,19,20,25]. A previous study has shown that being a single parent is inversely correlated with EOH [5], which is in line with our research that changes in the social environment or social support is a significant predictor for the maintenance of a reduction in EOH behavior. Likewise, studies have shown that convenience is positively correlated with EOH [5], and available time to prepare food at home is inversely correlated with EOH [10], which lends support to the construct of emotional transformation from MTM in positively influencing changes in EOH behavior. The differences between the Western US and the Midwest could be due to the ethnic makeup of the two regions. The reasons for the practice for change construct not being significant could be attributed to the lack of an intervention whereby participants are not exposed to a reflection on their EOH behavior. Health promotion interventions must operationalize significant constructs identified in this study to promote sustained in-home eating and reduce EOH behavior. 

### 4.1. Implications for Practice

This study found that the constructs of behavioral confidence and changes in the physical environment were significant in initiating changing EOH behavior to more in-home eating behavior. Educational interventions can be designed for different target audiences in different settings, for example, for adolescents in school settings, for young and middle-aged adults at worksites, for older adults at recreation centers, and so on. For building behavioral confidence, educational programs must use small steps to wean people from eating outside of the home. Strategies to lower cravings, resist pressures from friends and family, overcome inconvenience, overcome tight schedules, and other potential barriers must be built into educational interventions. To facilitate changes in the physical environment, cooking classes demonstrating in-home cooking, ensuring that all cooking supplies are available at home, and the home environment is conducive to cooking must be fostered.

This study found that the constructs of emotional transformation and changes in the social environment were significant in maintaining changing EOH behavior to more in-home eating behavior. For influencing emotional transformation, feelings associated with impulsive EOH should be directed into goals for cooking that will channel energy and reduce cravings for EOH. Further, self-motivation and overcoming self-doubt to be able to cook at home must be fostered by educational interventions. For mobilizing changes in the social environment, support from family members, friends, and social media must be utilized to sustain in-home eating. Educational programs and promotional messages can be designed to be delivered through social media, face-to-face interventions, online interventions, and hybrid interventions. These can be delivered at community recreation centers, faith-based organizations, universities/colleges, and other outlets.

There is also scope for policy-level interventions whereby health insurance companies and grocery stores can provide incentives for people to cook at home. More thought needs to go into operationalizing the construct of changes in the physical environment to influence people’s EOH behavior through policies.

### 4.2. Strengths and Limitations of Current Study

To our knowledge, this is the first theory-based study of EOH behavior using the fourth-generation MTM. This study developed a psychometrically robust instrument that can be used for future intervention studies. This study was able to provide empirical evidence in support of a highly predictive model in the form of MTM. However, there were some shortcomings of this study. This study utilized a cross-sectional design which, while being quick and inexpensive, failed to establish causality due to a lack of temporality. To address this limitation, future studies must test the MTM for EOH in experimental designs. Further, self-reports including intent for EOH instead of recording actual behavior were used in this study that lend themselves to measurement biases. While for attitudinal measures, self-report is the only approach; for actual behaviors, in future experimental studies, efforts must be made to measure these. Also, the test–retest reliability (stability) of the instrument was not tested, which is imperative for future experimental research. Next, our current sample size did not allow stratification analysis to unveil some possible dimensions of intersectionality, such as race, age, marital status, education, employment, etc., which could be an important recommendation for future studies with a larger sample size. Finally, residual confounding bias could have been introduced due to some variables being left unmeasured, such as ecologically conscious purchase behavior, and opting for healthy food choices were not investigated in this study [26,27].

## 5. Conclusions

The present study, the first of its kind, tested the role of MTM constructs in predicting EOH behavior. It was found that EOH behavior was very common in American households, with close to 75% of the participants reporting engaging in this behavior more than two times per week. This finding itself calls for nutritional interventions to curb this tendency. From a theoretical standpoint, two constructs each from the initiation and sustenance models of MTM were found to be highly predictive of the intent to change EOH behavior. These findings align with previous literature where behavioral confidence and changes in the physical environment are important for starting a behavior and emotional transformation, and changes in the social environment are important for maintaining the behavioral change of reducing EOH. There is a need to design educational interventions that promote in-home eating instead of frequent EOH for health, family bonding, economic, and other reasons. MTM is a robust model that can help in designing such interventions.

## Figures and Tables

**Table 1:** Quota constraints used in this study to mirror census representation.

Variable	Characteristics	Proportion in the Study Sample (%)	Census Distribution, Population Parameters * (%)
Gender	Female	50.6	50.5
	Male	49.4	49.5
Household income	Less than USD 19,999	21.2	21
	USD 20 K–50 K	35.6	35
	USD 50 K–100 K	35.6	35
	<USD 100 K	7.6	9
Age	18–34 years	32.9	33
	35–55 years	32.3	31
	55+ years	34.8	35
Ethnicity	Non-Hispanic White	61.9	60.1
	Non-Hispanic Black	12.3	13.4
	Hispanic	17.4	18.5
	Others	3.1	8
Region	Midwest	21.6	21
	Northeast	15.8	16
	South	41.2	41
	West	21.4	22

* https://www.census.gov/quickfacts/fact/table/US/PST045219 (accessed on 8 January 2024).

**Table 2:** Comparison of socio-demographic characteristics of the study groups (*n* = 532).

Variable Name	Categories	Overall Sample	Eat Outside of the Home (EOH) at Least Twice a Week		*p* Value	95% Confidence Interval
		*n* = 532	Group 1 Yes (*n* = 397)	Group 2 No (*n* = 135)		
Age in years (M ± SD)	-	45.71 ± 19.14	42.25 ± 17.78	55.89 ± 19.43	**<0.001**	44.08, 47.34
BMI	-	28.38 ± 7.00	28.57 ± 7.25	27.88 ± 6.26	0.31	27.76, 29.01
Gender	Male	261 (49.4)	196 (49.9)	65 (48.1)	0.7	44.73, 53.40
	Female	267 (50.6)	197 (50.1)	70 (51.9)	-	45.85, 54.52
Race/Ethnicity	Black or African American	71 (13.5)	62 (15.9)	9 (6.7)	**0.01**	10.57, 16.53
	White	324 (61.6)	227 (58.1)	97 (71.9)	**0.01**	56.61, 65.07
	Hispanic, Latino, Latina, or Latinx	94 (17.9)	72 (18.4)	22 (16.3)	0.55	14.52, 21.18
	Other (Alaska Native, American Indian, Asian, Multiracial, Others)	37 (7.0)	30 (7.7)	7 (5.2)	0.32	4.94, 9.46
Marital status	Married	192 (36.1)	144 (36.3)	48 (35.6)	0.92	32.00, 40.33
	Never married	166 (31.2)	135 (34.0)	31 (23.0)	**0.02**	27.29, 35.33
	Divorced/Separated/Widowed	127 (23.9)	83 (20.9)	44 (32.6)	**0.01**	20.31, 27.73
	Other	47 (8.8)	35 (8.8)	12 (8.9)	0.95	6.56, 11.57
Education	Graduate Degree	43 (8.1)	38 (9.6)	5 (3.7)	**0.03**	5.91, 10.73
	Professional Degree	23 (4.3)	22 (5.5)	1 (0.7)	**0.02**	2.76, 6.42
	College Degree (Associate or Bachelorate)	159 (29.9)	118 (29.7)	41 (30.4)	0.92	26.02, 33.98
	Some college but no degree	129 (24.2)	85 (21.4)	44 (32.6)	**0.01**	20.66, 28.12
	High school graduate (or equivalent including GED)	156 (29.3)	119 (30.0)	37 (27.4)	0.55	25.48, 33.39
	Less than a high school diploma	22 (4.1)	15 (3.8)	7 (5.2)	0.63	2.61, 6.19
Community type	Rural	153 (28.8)	106 (26.7)	47 (34.8)	0.16	24.95, 32.81
	Suburban	208 (39.1)	157 (39.5)	51 (37.8)	-	34.93, 43.39
	Urban	171 (32.1)	134 (33.8)	37 (27.4)	-	28.19, 36.30
Region	Midwest	115 (21.6)	88 (22.2)	27 (20.0)	0.62	18.19, 25.36
	Northeast	84 (15.8)	54 (13.6)	30 (22.2)	**0.02**	12.79, 19.17
	South	219 (41.2)	173 (43.6)	46 (34.1)	0.06	36.95, 45.48
	West	114 (21.4)	82 (20.7)	32 (23.7)	0.48	18.01, 25.16
Employment status	Yes	333 (62.6)	286 (72.0)	47 (34.8)	**<0.001**	58.33, 66.72
	No	199 (37.4)	111 (28.0)	88 (65.2)	-	33.28, 41.67
Religion	Christian	199 (37.4)	151 (38.0)	48 (35.6)	0.61	33.28, 41.67
	Non-Christian	333 (62.6)	246 (62.0)	87 (64.4)	-	58.33, 66.72
Median income	Less than USD 19, 999	111 (21.2)	73 (18.8)	38 (28.1)	0.10	17.49, 24.57
	USD 20,000–50,000	186 (35.6)	138 (35.6)	48 (35.6)	-	30.91, 39.18
	USD 50,001–100,000	186 (35.6)	145 (37.4)	41 (30.4)	-	30.91, 39.18
	Great than USD 100,000	40 (7.6)	32 (8.2)	8 (5.9)	-	5.43, 10.10
	No	376 (70.7)	273 (68.8)	103 (76.3)	-	66.61, 74.52

Group 1: Eat outside home (EOH) at least twice a week; Group 2: Do not eat outside home (EOH) at least twice a week. *p* values less than 0.05 are considered statistically significant and are bolded in the table. Percentages may not add up to 100% due to some missing data. If the overall *p* value of the group was significant (in more than 2 by 2 tables), then individual *p* values were calculated by the adjusted residuals method.

**Table 3:** History of eating and other behaviors among the study groups (*n* = 532).

Variable Name	Categories	Overall Sample *n* (%)	Eat Outside Home (EOH) at Least Twice a Week		*p* Value
		*n* = 532	Group 1 Yes (*n* = 397)	Group 2 No (*n* = 135)	
Currently smoke tobacco more than once a week	Yes	166 (31.2)	138 (34.8)	28 (20.7)	**0.002**
	No	366 (68.8)	259 (65.2)	107 (79.3)	-
Currently drink alcohol more than once a week	Yes	156 (29.3)	124 (31.2)	32 (23.7)	0.09
	No	376 (70.7)	273 (68.8)	103 (76.3)	-
When you do eat outside of the home, what kind of food do you prefer?	American	232 (43.6)	185 (46.6)	47 (34.8)	0.19
	Chinese	65 (12.2)	44 (11.1)	21 (15.6)	-
	Mexican	116 (21.8)	86 (21.7)	30 (22.2)	-
	Seafood	40 (7.5)	29 (7.3)	11 (8.1)	-
	Italian	31 (5.8)	21 (5.3)	10 (7.4)	-
	Others	48 (9.0)	32 (8.1)	16 (11.9)	-
How much money do you think your family spends on eating outside of the home per month?	USD 0–200	412 (77.4)	280 (70.5)	132 (97.8)	**<0.001**
	USD 201–400	70 (13.2)	67 (16.9)	3 (2.2)	**<0.001**
	USD 401–600	26 (4.9)	26 (6.5)	0 (0.0)	**<0.001**
	< USD 600	24 (4.5)	24 (6.1)	0 (0.0)	**<0.001**
Which meal do you eat outside home?	Breakfast	46 (8.6)	39 (9.8)	7 (5.2)	0.09
	Lunch	232 (43.6)	180 (45.3)	52 (38.5)	-
	Snacks	21 (3.9)	15 (3.8)	6 (4.4)	-
	Dinner	233 (43.8)	163 (41.1)	70 (51.9)	0.2
Where do you eat?	Fast-food restaurants	196 (36.8)	161 (40.6)	35 (25.9)	**<0.001**
	Restaurants	243 (45.7)	168 (42.3)	75 (55.6)	**0.01**
	Work (including canteens) and cafeterias	41 (7.7)	36 (9.1)	5 (3.7)	0.05
	Bars, friends’ or relatives’ houses, street, and others	52 (9.8)	32 (8.1)	20 (14.8)	**0.02**

Group 1: Eat outside home (EOH) at least twice a week; Group 2: Do not eat outside home (EOH) at least twice a week. *p* values less than 0.05 are considered statistically significant and are bolded in the table. Percentages may not add up to 100% due to some missing data. If the overall *p* value of the group was significant (in more than 2 by 2 tables), then individual *p* values were calculated by the adjusted residuals method.

**Table 4:** Mean scores and ranges of MTM constructs of changing eating outside home behavior (*n* = 532).

MTM Construct	Eat Outside of the Home (EOH) at Least Twice a Week		*p* Value	Mean Difference	95% CI of Mean Difference
	Yes (*n* = 397)	No (*n* = 135)			
Overall Initiation Score	1.94 ± 1.32	3.00 ± 1.25	**<0.001**	1.055	0.813, 1.297
Subscales					
Perceived Advantages	13.59 ± 4.93	12.66 ± 5.01	0.06	−0.933	−1.901, 0.036
Perceived Disadvantages	13.38 ± 4.56	12.30 ± 4.64	**0.02**	−1.074	−1.970, −0.178
Participatory Dialogue	0.21 ± 4.38	0.36 ± 5.32	0.78	0.141	−0.859, 1.142
Behavioral Confidence	14.26 ± 6.79	19.33 ± 6.79	**<0.001**	5.07	3.74, 6.39
Changes in the Physical Environment	7.17 ± 2.97	8.84 ± 3.11	**<0.001**	1.67	1.08, 2.26
Overall Sustenance Score	1.84 ± 1.24	2.75 ± 1.34	**<0.001**	−0.907	−1.154, −0.659
Subscales					
Emotional Transformation	6.67 ± 3.03	9.14 ± 3.22	**<0.001**	−2.47	−3.07, −1.87
Practice for Change	6.16 ± 3.05	7.87 ± 3.21	**<0.001**	−1.71	−2.32, −1.11
Change in the Social Environment	5.42 ± 3.22	6.04 ± 3.46	0.05	−0.62	−1.29, 0.04

*p* values less than 0.05 are considered statistically significant and are bolded in the table; all values are represented as means and standard deviation.

**Table 5:** Pearson correlations between MTM constructs used in this study (*n* = 532).

Variables	1	2	3	4	5	6
1. Participatory Dialogue	1	0.207 ** [0.124, 0.287]	0.161 ** [0.077, 0.243]	0.147 ** [0.063, 0.229]	0.143 ** [0.059, 0.225]	0.202 ** [0.119, 0.282]
2. Behavioral Confidence	0.207 ** [0.124, 0.287]	1	0.750 ** [0.711, 0.785]	0.786 ** [0.751, 0.871]	0.763 ** [0.725, 0.796]	0.501 ** [0.434, 0.562]
3. Changes in the Physical Environment	0.161 ** [0.077, 0.243]	0.750 ** [0.711, 0.785]	1	0.745 ** [0.705, 0.781]	0.692 ** [0.645, 0.734]	0.485 ** [0.417, 0.547]
4. Emotional Transformation	0.147 ** [0.063, 0.229]	0.786 ** [0.751, 0.871]	0.745 ** [0.705, 0.781]	1	0.741 ** [0.701, 0.777]	0.479 ** [0.411, 0.542]
5. Practice for Change	0.143 ** [0.059, 0.225]	0.763 ** [0.725, 0.796]	0.692 ** [0.645, 0.734]	0.741 ** [0.701, 0.777]	1	0.620 ** [0.565, 0.670]
6. Changes in the Social Environment	0.202 ** [0.119, 0.282]	0.501 ** [0.434, 0.562]	0.485 ** [0.417, 0.547]	0.479 ** [0.411, 0.542]	0.620 ** [0.565, 0.670]	1
Cronbach’s Alpha	-	0.910 [0.897, 0.921]	0.820 [0.791, 0.845]	0.874 [0.855, 0.892]	0.779 [0.744, 0.810]	0.728 [0.686, 0.766]
McDonald’s Omega	-	0.908 [0.897, 0.921]	0.822 [0.791, 0.845]	0.875 [0.855, 0.892]	0.781 [0.744, 0.810]	0.745 [0.686, 0.766]

Global Cronbach’s Alpha of the entire MTM survey = 0.926 [0.917, 0.935]; ** *p* < 0.01; “Participatory dialogue” was measured through “perceived advantages” (Cronbach’s Alpha = 0.797; McDonald’s Omega = 0.789) and “perceived disadvantages” (Cronbach’s Alpha = 0.743; McDonald’s Omega = 0.739).

**Table 6:** Hierarchical multiple regression for initiation of reducing EOH behavior (*n* = 532).

Variables	Model 1		Model 2		Model 3		Model 4	
	B	β	B	β	B	β	B	β
Constant	3.132 **		2.913 **		0.966 *		0.820	
Age	0.000	0.006	0.002	0.030	−0.004	−0.063	−0.005	−0.069
BMI	0.006	0.031	0.006	0.030	0.006	0.033	0.005	0.025
Gender: Male (Ref: Female)	−0.172	−0.066	−0.206	−0.079	−0.090	−0.035	−0.074	−0.029
Race: White (Ref: Black)	−0.035	−0.013	−0.009	−0.003	−0.049	−0.018	−0.060	−0.022
Hispanic	−0.319	−0.095	−0.348	−0.104	−0.168	−0.050	−0.161	−0.048
Other	0.155	0.028	0.241	0.044	0.219	0.040	0.208	0.038
Marital Status: Never Married (Ref: Married)	−0.100	−0.036	−0.080	−0.029	−0.063	−0.023	−0.049	−0.018
Divorced/Separated/Widowed	−0.197	−0.065	−0.165	−0.054	0.035	0.012	0.045	0.015
Other	−0.012	−0.003	−0.029	−0.006	0.124	0.026	0.155	0.032
Education: Professional Degree (Ref: Graduate Degree)	0.307	0.046	0.364	0.055	0.401	0.061	0.429	0.065
College degree	−0.030	−0.011	−0.019	−0.007	0.115	0.041	0.080	0.029
Some college/No degree	−0.215	−0.072	−0.214	−0.071	−0.055	−0.018	−0.098	−0.033
High school graduate	−0.077	−0.027	−0.032	−0.011	0.075	0.026	0.077	0.027
Less than high school	−0.095	−0.014	−0.083	−0.012	0.259	0.037	0.196	0.028
Community Type: Urban (Ref: Rural)	−0.100	−0.036	−0.044	−0.016	−0.005	−0.002	0.001	0.000
Suburban	0.031	0.012	0.064	0.024	0.038	0.014	0.038	0.014
Region: Northeast (Ref: Midwest)	0.188	0.053	0.238	0.067	0.010	0.003	0.036	0.010
South	0.070	0.027	0.096	0.037	0.000	0.000	0.007	0.003
West	0.098	0.031	0.075	0.024	−0.160	−0.051	−0.159	−0.050
Employment status: Working (Ref: Not Working)	0.209	0.079	0.197	0.074	0.168	0.063	0.171	0.064
Religion: Christian (Ref: Non-Christian)	−0.103	−0.038	−0.118	−0.044	−0.096	−0.036	−0.105	−0.039
Income: USD 20,000–50,000 (Ref: Less than USD 19,999)	0.101	0.037	0.115	0.043	0.099	0.037	0.076	0.028
USD 50,001–100,000	−0.139	−0.051	−0.070	−0.026	−0.032	−0.012	−0.040	−0.015
>USD 100,000	−0.191	−0.040	−0.151	−0.031	0.157	0.033	0.147	0.030
Do you eat outside of the home: Yes (Ref: No)	−1.185 **	−0.406	−1.150 **	−0.394	−0.653 **	−0.224	−0.661 **	−0.226
Do you currently use/smoke tobacco more than once a week? Yes (Ref: No)	0.045	0.016	0.086	0.030	0.077	0.027	0.070	0.025
Do you currently drink alcohol more than once a week? Yes (Ref: No)	0.028	0.010	0.038	0.013	0.009	0.003	−0.001	0.000
Participatory dialogue	-	-	0.056 **	0.202	0.016	0.060	0.016	0.057
Behavioral confidence	-	-	-	-	0.110 **	0.606	0.081 **	0.449
Changes in the physical environment	-	-	-	-	-	-	0.089 **	0.210
R^2^	0.168	-	0.206	-	0.495	-	0.513	-
F	3.338 **	-	4.132 **	-	15.043 **	-	15.579 **	-
ΔR^2^	0.168	-	0.038	-	0.289	-	0.018	-
ΔF	3.338 **	-	21.453 **	-	254.717 **	-	16.208 **	-

* *p*-value < 0.05; ** *p*-value < 0.001; Adjusted R^2^ of Model 4 = 0.480; initiation was measured through a single item: “How likely is it that you will reduce eating outside of the home to less than twice a week in the next week?”

**Table 7:** Hierarchical multiple regression for sustenance of reducing EOH behavior (*n* = 532).

Variables	Model 1		Model 2		Model 3		Model 4	
	B	β	B	β	B	β	B	β
Constant	3.768 **		1.354 *		1.214 *		0.853 *	
Age	−0.010 *	−0.143	−0.014 **	−0.210	−0.015 **	−0.216	−0.012	−0.168
BMI	0.006	0.032	0.010	0.050	0.009	0.047	0.010	0.053
Gender: Male (Ref: Female)	−0.196	−0.074	−0.120	−0.045	−0.129	−0.049	−0.064	−0.024
Race: White (Ref: Black)	−0.111	−0.041	−0.047	−0.017	−0.013	−0.005	0.022	0.008
Hispanic	−0.125	−0.037	0.073	0.021	0.133	0.039	0.168	0.049
Other	−0.117	−0.021	0.027	0.005	0.035	0.006	0.105	0.019
Marital Status: Never Married (Ref: Married)	−0.191	−0.067	0.008	0.003	0.001	0.000	0.057	0.020
Divorced/Separate/Widow	−0.255	−0.082	−0.119	−0.039	−0.034	−0.011	0.049	0.016
Other	−0.270	−0.055	−0.091	−0.019	−0.125	−0.026	−0.064	−0.013
Education: Professional Degree (Ref: Graduate Degree)	0.243	0.036	0.353	0.052	0.333	0.049	0.335	0.050
College degree	−0.168	−0.059	−0.092	−0.032	−0.035	−0.012	0.012	0.004
Some college/No degree	−0.321	−0.104	−0.247	−0.080	−0.181	−0.059	−0.195	−0.064
High school graduate	−0.321	−0.110	−0.239	−0.082	−0.224	−0.077	−0.208	−0.072
Less than high school	−0.473	−0.066	−0.142	−0.020	−0.194	−0.027	−0.283	−0.040
Community Type: Urban (Ref: Rural)	−0.033	−0.011	0.053	0.019	0.070	0.024	0.031	0.011
Suburban	0.053	0.020	0.068	0.025	0.069	0.025	0.050	0.019
Region: Northeast (Ref: Midwest)	0.125	0.034	−0.028	−0.008	−0.045	−0.012	−0.026	−0.007
South	−0.052	−0.019	−0.202	−0.075	−0.152	−0.057	−0.141	−0.053
West	−0.083	−0.026	−0.385 *	−0.120	−0.361 *	−0.112	−0.310 *	−0.096
Employment status: Working (Ref: Not Working)	0.074	0.027	0.044	0.016	0.008	0.003	−0.061	−0.022
Religion: Christian (Ref: Non-Christian)	−0.063	−0.023	−0.019	−0.007	−0.036	−0.013	−0.037	−0.013
Income: USD 20,000–50,000 (Ref: Less than USD 19,999)	0.183	0.066	0.181	0.066	0.139	0.050	0.103	0.037
USD 50,001–100,000	−0.136	−0.049	−0.044	−0.016	−0.083	−0.030	−0.046	−0.016
>USD 100,000	−0.455	−0.092	−0.090	−0.018	−0.118	−0.024	−0.151	−0.031
Do you eat outside of the home: Yes (Ref: No)	−1.180 **	−0.396	−0.629 **	−0.211	−0.609 **	−0.204	−0.600 **	−0.201
Do you currently use/smoke tobacco more than once a week? Yes (Ref: No)	0.226	0.078	0.287 *	0.099	0.254 *	0.088	0.228	0.079
Do you currently drink alcohol more than once a week?: Yes (Ref: No)	−0.057	−0.019	−0.096	−0.032	−0.089	−0.030	−0.095	−0.032
Emotional Transformation	-	-	0.249 **	0.605	0.167 **	0.406	0.156 **	0.378
Practice for change	-	-	-	-	0.111 **	0.263	0.045	0.106
Change in the Social Environment	-	-	-	-	-	-	0.113 **	0.281
R^2^	0.163	-	0.460	-	0.488	-	0.532	-
F	3.212 **	-	13.547 **	-	14.654 **	-	16.806 **	-
ΔR^2^	0.163	-	0.297	-	0.029	-	0.043	-
ΔF	3.212 **	-	245.193 **	-	25.129 **	-	41.015 **	-

* *p*-value < 0.05; ** *p*-value < 0.001; Adjusted R^2^ for Model 4 = 0.500; sustenance was measured through a single item: “How likely is it that you will reduce eating outside of the home to less than twice a week from now on?”

**Table 8:** Multinomial logistic regression to predict the likelihood of initiating a reduction in EOH behavior (*n* = 532).

Comparing Moderately Likely with Not at All or Somewhat Likely Combined						95% CI (LCL, UCL)	
Variables	B	SE	Wald	*p* Value	Odd Ratio	LCL	UCL
Age	−0.008	0.011	0.468	0.494	0.992	0.970	1.015
BMI	0.020	0.021	0.993	0.319	1.021	0.980	1.063
Gender: Male (Ref: Female)	−0.287	0.307	0.874	0.350	0.751	0.411	1.370
Race: White (Ref: Black)	0.031	0.449	0.005	0.944	1.032	0.428	2.487
Hispanic	−0.283	0.507	0.311	0.577	0.754	0.279	2.036
Other	1.262	0.769	2.693	0.101	3.532	0.782	15.943
Marital Status: Never Married (Ref: Married)	0.191	0.404	0.224	0.636	1.210	0.549	2.671
Divorced/Separate/Widow	0.255	0.415	0.380	0.538	1.291	0.573	2.909
Other	−0.632	0.614	1.060	0.303	0.532	0.160	1.770
Education: Professional Degree (Ref: Graduate Degree)	1.052	0.885	1.413	0.235	2.864	0.505	16.235
College degree	0.821	0.616	1.777	0.183	2.273	0.680	7.601
Some college/No degree	0.230	0.657	0.123	0.726	1.259	0.348	4.558
High school graduate	0.796	0.649	1.507	0.220	2.217	0.622	7.907
Less than high school	1.720	1.021	2.836	0.092	5.585	0.754	41.352
Community Type: Urban (Ref: Rural)	0.578	0.376	2.356	0.125	1.782	0.852	3.726
Suburban	0.223	0.364	0.375	0.540	1.250	0.612	2.552
Region: Northeast (Ref: Midwest)	0.498	0.517	0.928	0.335	1.646	0.597	4.539
South	−0.056	0.391	0.021	0.885	0.945	0.439	2.033
West	0.006	0.444	0.000	0.989	1.006	0.422	2.400
Employment status: Working (Ref: Not Working)	0.735	0.382	3.711	0.054	2.086	0.987	4.407
Religion: Christian (Ref: Non-Christian)	−0.288	0.301	0.919	0.338	0.750	0.416	1.351
Income: USD 20,000–50,000 (Ref: Less than USD 19,999)	0.525	0.432	1.476	0.224	1.690	0.725	3.941
USD 50,001–100,000	−0.059	0.464	0.016	0.899	0.943	0.380	2.341
>USD 100,000	0.941	0.670	1.970	0.160	2.561	0.689	9.525
Do you eat outside of the home: Yes (Ref: No)	−0.482	0.422	1.304	0.254	0.617	0.270	1.413
Do you currently use/smoke tobacco more than once a week? Yes (Ref: No)	−0.274	0.316	0.749	0.387	0.761	0.409	1.413
Do you currently drink alcohol more than once a week? Yes (Ref: No)	0.466	0.324	2.069	0.150	1.593	0.845	3.005
Participatory dialogue	0.067	0.035	3.673	0.055	1.069	0.998	1.145
Behavioral confidence	0.095	0.033	8.427	0.004	1.100	1.032	1.173
Changes in the physical environment	0.106	0.066	2.601	0.107	1.112	0.977	1.266
**Comparing Very or Completely Likely with Not at All and Somewhat Likely**						**95% CI (LCL, UCL)**	
**Variables**	**B**	**SE**	**Wald**	***p* Value**	**Odd Ratio**	**LCL**	**UCL**
Age	0.007	0.012	0.275	0.600	1.007	0.982	1.032
BMI	0.010	0.025	0.163	0.687	1.010	0.962	1.060
Gender: Male (Ref: Female)	−0.221	0.350	0.400	0.527	0.801	0.403	1.592
Race: White (Ref: Black)	−0.143	0.514	0.078	0.780	0.866	0.316	2.372
Hispanic	−0.043	0.576	0.006	0.940	0.958	0.310	2.961
Other	1.416	0.857	2.730	0.098	4.121	0.768	22.102
Marital Status: Never Married (Ref: Married)	0.344	0.468	0.540	0.462	1.411	0.564	3.531
Divorced/Separate/Widow	0.180	0.462	0.152	0.697	1.198	0.484	2.963
Other	0.658	0.623	1.116	0.291	1.931	0.570	6.547
Education: Professional Degree (Ref: Graduate Degree)	1.279	0.940	1.853	0.173	3.593	0.570	22.654
College degree	0.215	0.659	0.106	0.744	1.240	0.341	4.510
Some college/No degree	0.015	0.691	0.000	0.983	1.015	0.262	3.932
High school graduate	0.229	0.698	0.107	0.743	1.257	0.320	4.934
Less than high school	1.059	1.173	0.815	0.367	2.882	0.290	28.693
Community Type: Urban (Ref: Rural)	0.128	0.424	0.091	0.763	1.137	0.495	2.609
Suburban	0.032	0.397	0.006	0.937	1.032	0.474	2.248
Region: Northeast (Ref: Midwest)	0.129	0.578	0.050	0.823	1.138	0.366	3.537
South	−0.399	0.426	0.879	0.348	0.671	0.291	1.545
West	−1.085	0.517	4.413	0.036	0.338	0.123	0.930
Employment status: Working (Ref: Not Working)	0.865	0.419	4.271	0.039	2.376	1.046	5.398
Religion: Christian (Ref: Non-Christian)	−0.600	0.341	3.099	0.078	0.549	0.282	1.070
Income: USD 20,000–50,000 (Ref: Less than USD 19,999)	0.474	0.474	1.001	0.317	1.607	0.635	4.067
USD 50,001–100,000	−0.079	0.521	0.023	0.879	0.924	0.333	2.566
>USD 100,000	0.935	0.780	1.437	0.231	2.546	0.552	11.742
Do you eat outside of the home: Yes (Ref: No)	−1.345	0.428	9.876	0.002	0.260	0.113	0.603
Do you currently use/smoke tobacco more than once a week? Yes (Ref: No)	0.123	0.357	0.119	0.730	1.131	0.562	2.276
Do you currently drink alcohol more than once a week?: Yes (Ref: No)	−0.006	0.375	0.000	0.988	0.995	0.477	2.075
Participatory dialogue	0.067	0.038	3.178	0.075	1.070	0.993	1.152
Behavioral confidence	0.273	0.040	47.169	<0.001	1.314	1.216	1.421
Changes in the physical environment	0.290	0.080	13.088	<0.001	1.336	1.142	1.563

Note: Initiation was measured through a single item: “How likely is it that you will reduce eating outside of the home to less than twice a week in the next week?”

**Table 9:** Multinomial logistic regression to predict the likelihood of sustaining reducing EOH behavior (*n* = 532).

Comparing Moderately Likely with Not at All and Somewhat Likely						95% CI (LCL, UCL)	
Variables	B	SE	Wald	*p* Value	Odd Ratio	LCL	UCL
Age	−0.032	0.011	8.099	0.004	0.968	0.947	0.990
BMI	0.006	0.021	0.082	0.774	1.006	0.965	1.049
Gender: Male (Ref: Female)	0.188	0.316	0.355	0.551	1.207	0.650	2.241
Race: White (Ref: Black)	−0.180	0.466	0.148	0.700	0.836	0.335	2.085
Hispanic	0.334	0.544	0.376	0.540	1.396	0.480	4.058
Other	0.700	0.701	0.997	0.318	2.014	0.510	7.954
Marital Status: Never Married (Ref: Married)	−0.199	0.416	0.229	0.632	0.819	0.362	1.853
Divorced/Separate/Widow	0.952	0.434	4.817	0.028	2.590	1.107	6.060
Other	−0.150	0.590	0.065	0.799	0.861	0.271	2.734
Education: Professional Degree (Ref: Graduate Degree)	2.135	0.958	4.970	0.026	8.458	1.294	55.261
College degree	0.273	0.682	0.160	0.690	1.313	0.345	5.002
Some college/No degree	0.370	0.710	0.271	0.602	1.447	0.360	5.816
High school graduate	0.433	0.709	0.373	0.541	1.542	0.384	6.184
Less than high school	0.823	1.013	0.661	0.416	2.278	0.313	16.579
Community Type: Urban (Ref: Rural)	0.809	0.388	4.352	0.037	2.245	1.050	4.800
Suburban	0.432	0.373	1.341	0.247	1.540	0.742	3.196
Region: Northeast (Ref: Midwest)	−0.176	0.504	0.121	0.727	0.839	0.312	2.253
South	−0.351	0.388	0.816	0.366	0.704	0.329	1.507
West	−1.371	0.468	8.599	0.003	0.254	0.102	0.635
Employment status: Working (Ref: Not Working)	0.344	0.381	0.817	0.366	1.410	0.669	2.974
Religion: Christian (Ref: Non-Christian)	−0.253	0.302	0.698	0.403	0.777	0.429	1.405
Income: USD 20,000–50,000 (Ref: Less than USD 19,999)	0.539	0.437	1.520	0.218	1.715	0.728	4.042
USD 50,001–100,000	−0.110	0.476	0.054	0.817	0.896	0.352	2.277
>USD 100,000	−1.158	0.776	2.229	0.135	0.314	0.069	1.437
Do you eat outside of the home: Yes (Ref: No)	−1.184	0.409	8.383	0.004	0.306	0.137	0.682
Do you currently use/smoke tobacco more than once a week? Yes (Ref: No)	0.094	0.336	0.078	0.780	1.098	0.569	2.121
Do you currently drink alcohol more than once a week?: Yes (Ref: No)	0.323	0.332	0.948	0.330	1.381	0.721	2.646
Emotional Transformation	0.216	0.073	8.726	0.003	1.241	1.075	1.433
Practice for change	−0.017	0.076	0.047	0.828	0.984	0.848	1.141
Change in the Social Environment	0.247	0.063	15.605	<0.001	1.280	1.133	1.447
**Comparing Very or Completely Likely with Not at All and Somewhat Likely**						**95% CI (LCL, UCL)**	
**Variables**	**B**	**SE**	**Wald**	***p* Value**	**Odd Ratio**	**LCL**	**UCL**
Age	−0.032	0.012	6.746	0.009	0.968	0.945	0.992
BMI	0.014	0.025	0.318	0.573	1.014	0.965	1.066
Gender: Male (Ref: Female)	−0.331	0.355	0.866	0.352	0.718	0.358	1.442
Race: White (Ref: Black)	−0.197	0.526	0.140	0.708	0.821	0.293	2.303
Hispanic	0.605	0.620	0.953	0.329	1.832	0.543	6.181
Other	0.490	0.804	0.372	0.542	1.633	0.338	7.897
Marital Status: Never Married (Ref: Married)	0.300	0.460	0.426	0.514	1.350	0.548	3.326
Divorced/Separate/Widow	0.479	0.478	1.005	0.316	1.614	0.633	4.116
Other	−0.189	0.674	0.078	0.780	0.828	0.221	3.103
Education: Professional Degree (Ref: Graduate Degree)	1.107	1.038	1.139	0.286	3.026	0.396	23.130
College degree	0.015	0.681	0.000	0.983	1.015	0.267	3.855
Some college/No degree	−0.555	0.725	0.588	0.443	0.574	0.139	2.374
High school graduate	−0.631	0.732	0.744	0.388	0.532	0.127	2.233
Less than high school	−0.943	1.196	0.622	0.430	0.389	0.037	4.061
Community Type: Urban (Ref: Rural)	0.419	0.433	0.937	0.333	1.520	0.651	3.552
Suburban	0.214	0.402	0.284	0.594	1.239	0.563	2.723
Region: Northeast (Ref: Midwest)	−0.248	0.553	0.201	0.654	0.780	0.264	2.308
South	−0.743	0.445	2.779	0.095	0.476	0.199	1.139
West	−1.367	0.515	7.049	0.008	0.255	0.093	0.699
Employment status: Working (Ref: Not Working)	0.015	0.413	0.001	0.972	1.015	0.452	2.278
Religion: Christian (Ref: Non-Christian)	−0.351	0.337	1.087	0.297	0.704	0.364	1.362
Income: USD 20,000–50,000 (Ref: Less than USD 19,999)	0.485	0.485	1.001	0.317	1.624	0.628	4.202
USD 50,001–100,000	−0.124	0.536	0.054	0.816	0.883	0.309	2.525
>USD 100,000	−0.350	0.771	0.206	0.650	0.705	0.155	3.195
Do you eat outside of the home: Yes (Ref: No)	−1.733	0.433	15.997	<0.001	0.177	0.076	0.413
Do you currently use/smoke tobacco more than once a week? Yes (Ref: No)	0.655	0.371	3.123	0.077	1.925	0.931	3.979
Do you currently drink alcohol more than once a week? Yes (Ref: No)	−0.201	0.379	0.281	0.596	0.818	0.389	1.720
Emotional Transformation	0.564	0.088	41.279	<0.001	1.758	1.480	2.089
Practice for change	0.086	0.082	1.110	0.292	1.090	0.929	1.279
Change in the Social Environment	0.383	0.067	33.008	<0.001	1.467	1.287	1.672

Note: Sustenance was measured through a single item: “How likely is it that you will reduce eating outside of the home to less than twice a week from now on?”

## Data Availability

The data presented in this study are available on request from the corresponding author. The data are not publicly available due to ethical reasons.
